# Adenoviral Producer Cells

**DOI:** 10.3390/v2081681

**Published:** 2010-08-16

**Authors:** Imre Kovesdi, Susan J. Hedley

**Affiliations:** VectorLogics, Inc., 550 11th Street South, Birmingham, AL35294, USA; E-Mail: ikovesdi@vectorlogics.com

**Keywords:** producer cell lines, adenovirus, Ad vectors, RCA

## Abstract

Adenovirus (Ad) vectors, in particular those of the serotype 5, are highly attractive for a wide range of gene therapy, vaccine and virotherapy applications (as discussed in further detail in this issue). Wild type Ad5 virus can replicate in numerous tissue types but to use Ad vectors for therapeutic purposes the viral genome requires modification. In particular, if the viral genome is modified in such a way that the viral life cycle is interfered with, a specific producer cell line is required to provide trans-complementation to overcome the modification and allow viral production. This can occur in two ways; use of a producer cell line that contains specific adenoviral sequences incorporated into the cell genome to trans-complement, or use of a producer cell line that naturally complements for the modified Ad vector genome. This review concentrates on producer cell lines that complement non-replicating adenoviral vectors, starting with the historical HEK293 cell line developed in 1977 for first generation Ad vectors. In addition the problem of replication-competent adenovirus (RCA) contamination in viral preparations from HEK293 cells is addressed leading to the development of alternate cell lines. Furthermore novel cell lines for more complex Ad vectors and alternate serotype Ad vectors are discussed.

## 1. Producer cells for non-replicating first generation Ad vectors

**Figure 1.**
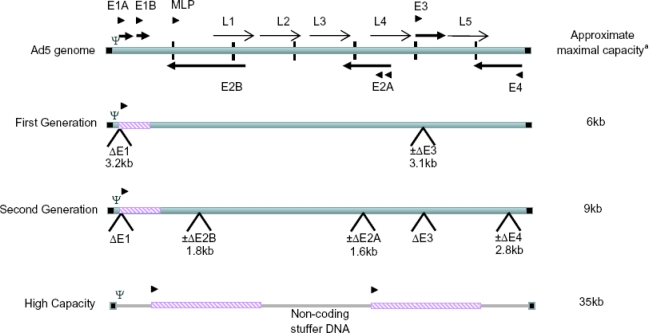
Genome structures of first and second generation and high capacity adenovirus vectors. Promoters are indicated by black triangles, early and late transcription units are indicated by thick and thin arrows respectively. The inverted terminal repeats (ITR) are indicated by black squares and the packaging signal, Ψ is indicated. Non-adenoviral sequences in the high capacity genome are indicated by a thin line, while transgenes in all genomes are indicated by diagonal striped boxes. ^a^A very large number of different systems are in use with specific extra capacity. For second generation vectors deletion of 3 gene loci and a conservative 103% genome size was used for these estimates.

Many therapeutic Ad5 vectors are non-replicating (or replication deficient) whereby the genome is deleted in the E1 region often in combination with the E3 region to provide space for alternate gene expression cassettes (first generation Ad vectors, see Figure 1). The E1 region encodes proteins necessary for the expression of the other early and late genes, hence initiating the viral life cycle. Therefore when the E1 region is replaced with an expression cassette to produce the gene product that is useful in therapy, such as suicide genes, antigens and antibodies, a producer cell line containing adenovirus E1 sequences is required to complement for this region. The E3 region, which encodes products that counteract host defense mechanisms, however is dispensable and not essential for viral replication *in vitro*, so it is not necessary to trans-complement for E3 [1].

To trans-complement for the lack of E1 the historic cell line has been HEK293 [2,3] (Table 1). The human embryonic kidney (HEK) 293 cell line was developed over 30 years ago through an insertion of E1A and E1B sequence [2], from nucleotides (nt) 1 to 4344, into chromosome 19 at 19q13.2 [4].

**Figure 2.**
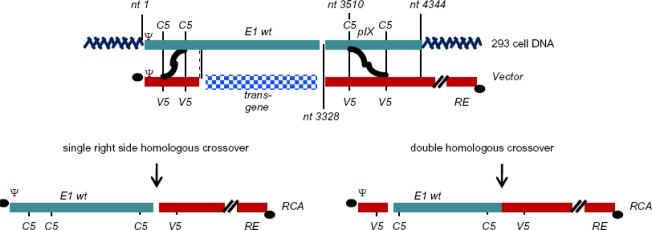
RCA generation in HEK293 cells. The complementary regions of Ad5 sequence in the vector and the HEK293 genome are aligned at the top of the figure. These regions allow for homologous recombination to occur and for the rescue of recombinant competent adenovirus.

The disadvantage with the HEK293 cell line is the potential for the generation of replication-competent adenovirus (RCA) contamination within the viral preparation. This can occur because many first-generation Ad vectors are deleted from nt 400 to 3500 and thus still retain significant homology with the cellular DNA allowing for a double crossover recombination event to take place [15] (Figure 2). While the occurrence of RCA is very low in the first passages of the virus [16], this is problematic for large-scale viral production and clinical applications [15–19] due to safety issues in a therapeutic product. In 2001, the FDA established that replication-defective adenoviral vector preparation contain less than 1 RCA in 3 × 10^10^ viral particles (Biological Response Modifiers Advisory Committee). Despite this issue HEK293 was and still remains a major host cell line for Ad vector development including development of master cell banks and development and optimization of Ad-manufacturing processes [20].

In the 90s, alternate E1 producer cell lines were being evolved (Table 1). One of the first was the 911 cell line generated by incorporation of Ad5 nt 79 – 5789 into the genome of human embryonic retinoblasts (HER) cells [5] through plasmid transfection. The 911 cell line was determined to outperform HEK293 in plaque formation and attainment of yields and consequently became another favored cell line. Despite the attractiveness of these features, the level of RCA contamination remained similar to that of HEK293. Around the same time, a cell line complementing E1 was developed from A549 lung carcinoma cells [6]. This cell line had incorporated a reduced Ad5 sequence (nt505–4034) under the regulation of a non-Ad promoter, phosphoglycerate kinase (PGK). However the production of virus in this cell line was poor, although it is unclear whether this is due to a 1bp deletion in the E1B region, abolishing expression of the E1B-55kDa protein or another reason. Despite this, the overall strategy of using smaller Ad5 E1 sequences led to the development of the PER.C6 cell line [7].

**Figure 3.**
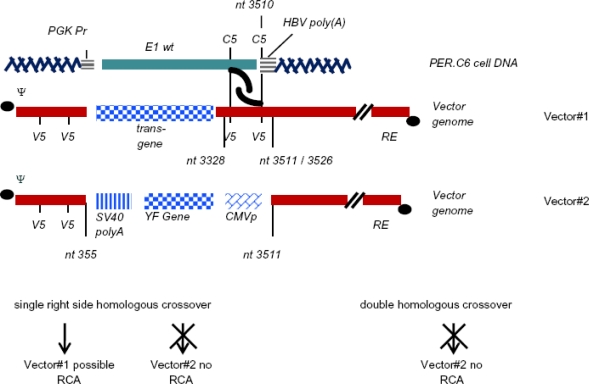
Effect of vector design on RCA free production in PER.C6 cell line. In Vector #1 there is a short stretch of homology with the PER.C6 Ad5 sequence that allows homologous recombination. This results in atypical RCA. When the vector has no homology (Vector #2) no homologous recombination occurs and the viral production is RCA free.

Like the 911 cell line, PER.C6 is also based on HER cells. The Ad5 sequence incorporated into the cell genome was the E1A- and E1B encoding sequences, nt 459–3510, under control of the human PGK promoter to compensate for the removal of the 5′ endogenous E1A promoter. Furthermore, the coding region for the minor capsid pIX protein which is in the 3′ region following the E1B 55-kDA coding region was also deleted in this construct. The smaller fragment of Ad5 sequence thus incorporated into the cell line has limited homology to many E1-deficient adenoviral vectors and double homologous recombination is not possible when using an appropriately designed Ad vector, thereby eliminating RCA. However, further studies indicated that rare unconventional recombination can still occur if the Ad vector genome is not perfectly matched. There only needs to be a short stretch of homology between cellular DNA and the vector genome to allow recombination [15] (Figure 3) resulting in atypical or pseudo RCA. It was determined that while the particle does contain intact E1 sequences, the virus cannot replicate on its own as genomic deletions also occur elsewhere in the vector and have been termed helper-dependent E1-positive (HDEP) viral particles [15,21]. These factors indicate that the design of the Ad vector still requires consideration and removal of any overlapping fragments to preclude these events from occurring even when using PER.C6 cells. However, PER.C6 is probably the best designed and most favorable cell line for the development of first generation Ad5 vectors for clinical development to date. PER.C6 master cell banks for cGMP vector production have been established and assessed (reviewed [22]). A major disadvantage to this cell line is that unlike HEK293 which is more freely available to the scientific community, PER.C6 currently has strict licensing costs/uses preventing many laboratories from working with this cell line. Furthermore, the PER.C6 line is considered less robust than HEK293 cell line as it is less adaptable to serum free culture for large scale production Ad vectors.

Due to the RCA issue and potential licensing issue several other laboratories have developed E1 complementary cells that contain smaller adenoviral E1 DNA segments than HEK293 cells and can propagate vectors to high titers similar to those seen on HEK293 [8–11] (Table 1). All of these cell lines have reduced or eliminated sequence homology between cellular DNA and viral genome to prevent the opportunity of the double homologous recombination crossover occurring. These cell lines are based on human primary amniocytes, human embryonic lung HEL 299 cells or HeLa with the incorporated E1A region driven by an alternate promoter (as detailed in Table 1). Unfortunately, the HeLa cell line is not allowed for commercial use because of its high tumorigenicity.

The requirement of the pIX sequence in first generation Ad vector producer cells has been questioned [6,8]. Of the more recent cell lines that contain contiguous E1A/E1B sequence, only PER.C6 and UR lack pIX sequences. The presence of partial pIX sequences in other cell lines could potentially influence RCA, however all have been reported to have significant reduction of RCA [8–10] as a non-homologous recombination is required at the 5′ end With respect to future cell lines being developed pIX sequence probably should be excluded. pIX has been described to have many different roles (reviewed [23,24]), including genome packaging [25,26] leading to infectious/non-infectious particles [26] and as a transcriptional activator [27,28]. It is thought that pIX is dispensable for viral replication [27,28] although this may only be the case in HEK293 rather than cell lines lacking pIX sequences such as PER.C6 [24,29]. The major issue though with pIX is that it is a minor capsid protein, and while the absence of pIX does not affect capsid assembly thermostability is significantly reduced due to lack of capsid incorporation [30]. Most importantly then pIX is required for stabilizing the capsid and providing temperature resistance that is needed for commercial manufacture. Therefore it is essential that pIX be expressed at a high enough level for capsid incorporation to achieve a commercially viable vector. In the case of first generation Ads this is preferable from the vector, although vectors further deleted in the pIX region could be trans-complemented by pIX expressing cell lines to increase expression to a level that allows pIX to be capsid incorporated [31,32].

Apart from the HEK293 cell line which was generated with sheared Ad5 DNA, the main strategy for generating producer cell lines for E1 trans-complementation has involved insertion of the contiguous sequence of E1A and E1B into the cell line genome utilizing DNA plasmid based systems. However, new and novel approaches are being taken to reduce and eliminate RCA, while maintaining trans-complementation. One approach utilized adenoviral E1A and E1B gene integration into the A549 genome at separate locations [13]. This was achieved using co-infection of cells with two retroviral vectors, one for each gene. Two of the stable clones attained, Ac51 and Ac139 were shown to be E1 complementary and support production of E1-defective adenoviruses without RCA generation. Furthermore the titer levels attained matched those seen in HEK293 and PER.C6 cells. In a similar strategy a producer cell line, SL0003, was generated through sequential incorporation of E1A and E1B genes into A549 cells [14]. Rather than use retroviral vectors for delivery, this strategy utilized DNA plasmids. As with Ac51 and Ac139, SL0003 could support high titer level production without the generation of RCA.

**Figure 4.**
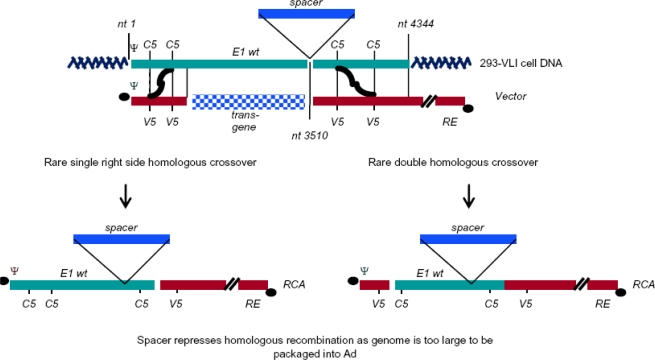
RCA free vector production in 293-VLI Cells. An ∼8 kb sequence of DNA containing advantageous genes, the spacer, will be incorporated into the HEK293 Ad sequence at nt3510. While this does not prevent homologous recombination due to retention of homology between the genome and Ad vector, packaging of the Ad vector will be suppressed due to the increased size.

A unique approach is being explored in our own laboratory as an alternate means to overcome RCA. In this instance, the Ad5 sequence in HEK293 cells would be modified to contain a large ∼8 kb spacer sequence of DNA, inserted at nt3510 (Figure 4). To achieve this modification, the spacer plasmid is designed to contain homologous Ad5 E1 sequences around the spacer sequence to allow for homologous recombination into the HEK293 genome. The insertion of spacer DNA following nt 3510 would still allow trans-complementation of the E1A/E1B functions in E1 deleted Ad vectors, but should homologous recombination between the Ad5 vector and the modified HEK293 genome occur, the Ad5 vector genome would become too large to be packaged (Figure 4). Ad5 vector genomes up to 105% than the normal genome size can still be packaged successfully [33], whereas in this case the genome size would ∼114% larger than normal. One concern though is that the Ad genome could undergo rearrangements resulting in smaller replication competent genomes which could be packaged [33] and this would have to be carefully assessed once the cell line is established. We are currently generating the new cell line with a spacer that also contains functional cassettes expressing advantageous genes which would potentially allow the new cell line to have a dual purpose of high levels of Ad vector and protein production [12].

First generation Ad vectors are the workhorse vector for many vaccine and gene therapy strategies, although they do have limitations as discussed in the following sections. The E1 complementing packaging cell lines developed to produce these vectors form the basis for all cell lines utilized with other types of genome deleted Ad vectors. The most important aspect to arise from this work is overcoming RCA, and this relies not only on how much of the Ad5 E1 sequences are incorporated into the cell line genome, but also careful design of the vector genomes themselves. Therefore the future for new cell line development is the incorporation of advantageous genes into the genome to improve productivity, adaptability and robustness as described for the 293-VLI model.

## 2. Producer cells for non-replicating second generation Ad vectors

Second generation Ad vectors contain further genome deletions, in the E2 and/or E4 regions (see Figure 1). The E2 region encodes three essential proteins controlling viral replication, the DNA-binding protein (DBP) transcribed from the E2A region, and terminal protein and viral DNA polymerase from E2B (reviewed [34]). E4 products are essential for productive virus infection but of the several possible open reading frame (ORF) products (reviewed [35]) encoded from this region it has been determined that only one, either ORF3 or ORF6 is absolutely required for successful viral growth in tissue culture [36–39]. Removal of either of E2 of E4 will inactivate de novo synthesis of viral proteins involved for viral DNA replication. Even though E1 proteins are removed from first generation vectors, delivery of high titers of E1 deleted vectors, and the presence of E1-like factors in many cells can allow for expression of other viral proteins [40–42]. These viral proteins would normally initiate a strong immune response dampening the efficacy of a first generation Ad vector [43–45]. Apart from lowering the immune response and thus enhancing vector safety, an additional benefit of removal of these genes is that larger expression cassettes can be incorporated into the genome, increasing the size from ∼6 kb to ∼9 kb.

In the early-80s and early-90s it was demonstrated that E2 defective Ad vectors [46,47] and E4 defective Ad vectors [48] could be propagated in trans-complementing cell lines based on HeLa and Vero cells respectively. Since then a wide array of cell lines, based on HEK293, 911 and E1 modified A549 cells, have been generated to allow propagation of Ad vectors lacking genes from the E2 and/or E4 regions in addition to the standard E1 deletion (Table 2) [31,49–61]. One of the main differences from E1 complementing cell lines, where E1A and E1B are expressed constitutively, is that several of these cell lines rely on the use of conditionally active transcription units or gene products to control the expression of these proteins as the viral products from E2A and E4 regions are toxic to the cells. For example, it was shown that expression of high levels of E2A viral product in the presence of E1 proteins is toxic to cells [46].

E2 complementing cell lines either express E2A (DBP) or E2B (Ad DNA polymerase and/or precursor terminal protein) viral products. DBP is required to be expressed at high levels for successful viral replication and therefore most E2A complementing cell lines are prepared with controlling elements. The 293-C2 line [56] was constructed with constitutive gene expression, but this group eventually developed an alternate cell line, E2T, relying on tetracycline controlling elements [61]. The tetracycline system relies on two cassettes stably inserted into the genome, the transactivator protein (tTA) cassette and the *tet* operator sequences in the CMV promoter region of the E2A cassette. In the presence of tetracycline, E2A is not expressed, but in the absence E2A is expressed. Viral production in 293-C2 is about 10- to 30- fold below those of E2 wild type vectors [56], while E2T in the absence of tetracycline produced viral yields of E2A deleted Ad vectors similar to that of wild E2 vectors [61]. Other E2A cell lines, 293-E2A [59] and AE1-2a [53] rely on glucocorticoid-responsive elements such as the mammary tumor virus promoter (MMTV) long terminal repeat and mouse mammary tumor virus promoter (pMAM) respectively, with expression induced by dexamethasone. Growth kinetics of an E1/E2A Ad vector in 293-E2A was similar to that of a comparative first generation Ad vector, but infectious particles were reduced by 5- to 30- fold under this system [59]. In the AE1-2a cell line, a delay in cytopathic effect was seen with an E1/E2A/E3 deleted Ad vector yet infectious particles yields were comparable levels to a first generation Ad vector in this instance [53].

It is known that E2B mRNA is expressed at low levels in Ad-infected cells [62] and therefore it is thought that cell lines expressing low levels of these viral products will be more appropriate in complementing deletions in Ad vectors. With this in mind two main cell lines have been generated. The C7 cell line was sequentially generated to express Ad2 140kDa DNA polymerase protein [50] and the precursor terminal protein (pTP) [57]. Both genes are constitutively expressed under the Rous sarcoma virus-long terminal repeat promoter element and cell growth and viability was not affected. Viruses containing mutations in the E2B region were successfully complemented by the C7 cell line to permit viral propagation at levels similar to E2B wild type vectors. 293-pTP, complementing only for the pTP uses a tetracycline regulatory system, wherein the cell line contains the tetracycline repressor/VP16 transactivator protein (tTA) and the pTP is under control of a tetracycline-dependent promoter [54]. This system was found to improve cell growth and viability over a previous version of 293-pTP which constitutively expressed pTP [63]. These tetracycline controlled 293-pTP cells were shown to efficiently complement a temperature sensitive pTP Ad vector when the cells expressed high, inducible levels of pTP.

Initial studies with E1/E4 deleted vectors utilized the incorporation of full length E4 into cell lines [31,49]. VL2-20 and VK10-9 cells, containing full length E4 controlled by dexamethasone inducible MMTV promoter, were permissible to propagation of an E4 deleted vector, but a delay in CPE was seen and 10-fold lower yields obtained [31]. However another full length E4 expressing cell line was reported, with the E4 cassette under the control of a mouse alpha inhibin promoter containing a cAMP response element that demonstrated yields of E1/E4-deleted vectors attained were comparable to E1-deleted vectors [49]. In 1996, two groups developed E4-ORF6 cell lines confirming that only one E4 open reading frame is required to complement for the E4 region. These cell lines, 293-ORF6 [51] and MT-ORF6 [52], were based on HEK293 cells with ORF6 under the control of a zinc inducible sheep metallotheinen promoter. In MMTV-ORF6 [52] the E4 ORF6 was under the control of the MMTV promoter as the inducible elements. An E1/E4 deleted Ad vector was reported to propagate with the MT-ORF6 and MMTV-ORF6 lines [52] but yields were not compared with an E1 deleted vector. In 293-ORF6 cells, yields of E1/E4 deleted Ad vector were equivalent to E1 deleted vector levels [51]. Even though these E4-ORF4 expressing cell lines were reported to successfully complement the deletion of E4, several other full length E4 and E4-ORF6+7 expressing cell lines were generated from 1996 onwards [55,58–60]. IGRP2 [55], with ORF6+7 under the control of the MMTV long terminal repeat, 911E4 [58] under a tetracycline system, and 293-E4 [59] under E1A homologous inducible E4 promoter and 294-E4ORF6+7 [59] under a tetracycline system all showed similar results in that yield of E1/E4 deleted vectors were reduced. However, A70.S54, a cell line complementing for triple deleted vector (E1/E2A/E4) was able to produce this vector as comparable levels to wild-type E4 vectors [60]. Some of the differences seen in the yields and viral growth are due not only to the cell line utilized but also to the differences in the vector design. For example, a small insert placed in the deleted E4 region can increase production levels by as much as 30 fold [51].

Despite extensive development of second-generation Ad vectors and cell lines throughout 1995 to 2000 many of these vectors have faded into the background. Yields of these vectors can be reduced when compared to respective first generation vectors, depending on the cell line and vector system used. Furthermore comparative studies between E2A or E4 and their respective isogenic E1 deleted vectors indicate that E2A deletions confer limited improvement [59,64,65] while the results are more controversial with respect to E4 deletions [52,59,66,67]. Although attenuated toxicity towards the vectors can be seen, stable transgene expression is generally not seen. These vectors have therefore been superceded by high capacity Ad vectors (HC-Ad) for long-term transgene expression (reviewed [68]). However, several of the producer cell lines have been utilized for other purposes as seen in the next two sections, but only the second generation producer line, 293-ORF6 [51] has been used in GMP productions.

## 3. Producer cells for high capacity Ad vectors

HC-Ad vectors, the so-called “gutted” Ads form the third class of non-replicative Ad vectors (see Figure 1). With these vectors, all the viral genes are deleted leaving only the cis-acting sequences necessary for viral DNA replication and packaging, allowing for large therapeutic gene incorporation or multiple genes. Furthermore, apart from pre-existing immunity, additional immune response to de novo viral protein production is essentially removed. These vectors when used *in vivo* result in long-term, high-level transgene expression combined with negligible toxicity (review [68]). The main disadvantage for these vectors is the complex production due to the need not only for a suitable producer cell line, but also a complementing Ad vector, so-called helper Ad vector (HAd), which provides the necessary packaging proteins of the viral particle. Thus far the process has not been simplified to just the HC-Ad and a producer cell line that can trans-complement for the entire Ad genome, as there is likely toxicity associated with expression of all the viral proteins within the cell [60]. The requirement of the HAd brings about the problem of contamination from the HAd when using E1 complementing producer cells. Several methodologies have been developed to construct HC-Ads (review [68]) but the predominant system involves Cre recombinase or a similar recombinase.

To reduce contamination of HAd a helper-dependent system was developed that involved generating a cell line expressing Cre recombinase, 293Cre4, in combination with modifying the HAd to contain *loxP* sites flanking the packaging site [69]. Due to the expression of Cre recombinase in the producer cell line the packaging signal is excised thus preventing packaging of the helper virus DNA. Several comparable Cre- [70–75] and/or FLP- [76,77] based systems have been designed based on the 293Cre4 Cre/Lox system. There are fundamental differences between the HAd, HC-Ad, cell lines and protocols between laboratories, however vector design and general protocol discussion is outside of the realm of this particular review. With respect to the producer cell lines, Cre or FLPe recombinase expressing lines were derived from E1 producer cell lines such as HEK293 [70,77–79] and PER.C6 [75], or 293 producer cells complementing E2A, E2T [72] and C7 [73] (Table 3). Generally the introduction of the Cre or FLPe recombinase cassette was through transfection of the appropriate plasmid. In the case of the development of PER.C6-Cre, this was conducted using a retroviral vector.

It should be noted that even with this type of system, contamination is not fully eliminated (0.1% to 1% is usually seen), but physical separation of the HC-Ad and HAd through CsCl ultracentrifugation can address this issue. For small preparations this is generally acceptable, however for industrial-scale clinical grade vector production further measures have to be taken [78], especially as some of this contamination can contain packaging competent HAd. Palmer & Ng, 2003 [78] have described further modifications to the HAd as means to improve the contamination issue by reversing the packaging signal with respect to the HC-Ad thus rendering the recombinant genomes too large to be packaged if homologous recombination occurs [80]. RCA is still potentially an issue with these vectors and therefore PER.C6 cells expressing Cre is a useful cell line to prevent this [75]. Another issue with the production of HC-Ad is that low titer levels are usually attained due to using adherent cell cultures. The 116 Cre expressing cell line was derived from a subclone of HEK293 cells, 293N3S [81] which can be cultured in suspension and this greatly enhances the titer levels [78], as has been seen with suspension cultures of PER.C6-Cre [75]. Even though these vectors are complex to produce, their increased cassette incorporation, improved expression and lower immunity issues drive their development.

## 4. Producer cell lines for novel Ad vector serotypes

Within the human population there are high titers of pre-existing neutralizing antibodies against Ad5 and Ad2 serotypes [82–85] due to the general exposure to Ads. Therefore while most research and development of Ad vectors has utilized the Ad5 and Ad2 serotypes, efficacy of these vectors can be severely compromised [86–89]. An additional observation is that upon re-administration liver toxicity is increased [90], and in human blood neutralizing antibodies may activate complement and induce inflammatory reactions [91]. In an attempt to circumvent this issue Ad vectors derived from different human [83,84,92–104] and animal [105–110] serotypes, to which the human population has a lower prevalence of neutralizing antibodies, are currently being investigated. Non-replicating versions of alternate serotypes face similar issues to the first-, second generation and HC-Ads based on Ad5 serotype, regarding which producer cell line is suitable to achieve production and propagation of the vector.

Human subgroup B adenoviruses, in particular, Ad11 and Ad35 are emerging as strong contenders for replicating defective vectors [83,84,93,95,96,98,100]. E1-deleted Ad35 and Ad11 vectors cannot be propagated on regular E1 producer cell lines, although E1A-only deleted Ad35 can be propagated on PER.C6 [83]. Therefore two initial approaches were taken to propagate E1-deleted vectors. One utilized existing second generation producer cell lines following the observation that Ad7 vectors (another subgroup B adenovirus) could be propagated in the E4 complementing cell line, 293-ORF6 [92,94]. VK10-9 which complements E1/E4 deletions [31] produced yields of Ad35 that were slightly lower yields than that seen with Ad5 [95], and A70.S54 [60], a triple deletion complementing cell line (E1/E2A/E4), was also used [83] albeit producing lower yields than an E1A deleted vector.

In the second approach new packaging cell lines CRE35G3 [93] and PER.C6/55K [84] that express Ad5 E1 and Ad35 E1B-55kDa proteins have been derived. These cell lines allow successful propagation of E1-defective Ad35 vectors to similar viral titers as Ad5. An Ad11 packaging cell line was also developed, 293-Ad11-E1B55K, which was produced by transfecting and generating a stable clone from 293 cells with a plasmid containing the Ad11 E1B55K gene under the control of human PGK [98]. This E1B55K packaging line approach has also been utilized for the construction of another subgroup B serotype, E1-deleted Ad3 in which 911 cells were modified to express Ad3 E1B55K gene [97]. While production yields of these defective viruses match those of Ad5, it has been noted that Ad35 and Ad11 vectors have increased viral particle to infectious particles compared with Ad5 when propagated on the respective E1B55K lines. This effect is attributed to cellular receptor properties as subgroup B viruses such as Ad3, Ad11 and Ad35 use CD46 receptor for infection rather than CAR that all other Ad serotypes use [111–113]. One important advantage demonstrated from the studies with Ad35 and Ad11, is that replication-competent Ad35 or Ad11 is absent in the viral preparations due to lack of homology between the genomic DNA of the virus and the E1B55K packaging cell lines.

Recently a new strategy for Ad35 has been developed relying on further modification of the Ad vector genome [100]. It has been reported that the Ad5 E1B55kDa protein forms a complex with E4 ORF6 proteins to increase selective export of late viral mRNA [114–116]. The observation that E1-deleted Ad35 vectors only propagate in cells lines expressing either E4-ORF6, or Ad35 E1B55K indicate for successful viral replication to occur these proteins should derive from the same subgroup. Therefore, E1-deleted Ad35 vectors have further been modified to have the E4 region replaced with Ad5 E4-ORF6 and this permits viral propagation on unmodified PER.C6 cells [100] whereas previously only E1A-deleted vectors could be propagated on this cell line. The main benefit of modifying the vectors this way allows for propagation on well established and regulatory approved cell lines resulting in one less step when moving the novel Ad vector itself through regulatory approval. This E4 serotype switching methodology has also been applied to the development of a subgroup D serotype Ad49 vector [101,103] as well as to recombinant Ad vaccine vectors derived from several subgroup B (Ad11, Ad50) and subgroup D viruses (Ad26, Ad48) [103]. Other human serotypes that have been utilized however can be propagated in E1 producer cells and E1/E4 producer cells. This includes a different subgroup D serotype, Ad19a that was E1/E3-deleted and propagated to wt levels in HEK293 cells [102], a subgroup C serotype Ad6 based vaccine that can be propagated on PER.C6 cells [99] and a subgroup F serotype, Ad41 which when E1 deleted can still be propagated on 293-ORF6 cells [104].

Several non-human serotypes derived from bovine [106], canine [105,107] and simian [108–110,117] sources have been developed as potential E1-deleted Ad vectors for use as gene delivery or vaccine vectors for human therapies. In the case of the bovine, BAV-3 and canine, CAV-2 vectors, specialized E1 producer cells based on appropriate species cell lines, such as bovine kidney and fetal retinal cells expressing Ad5 E1 sequences [106] and canine kidney cells expressing the CAV-2 E1 [105], have been developed to package and propagate these vectors. The chimpanzee derived Ad vectors of subgroup E can be propagated on HEK293 cells and therefore can be grown under standard methods [108,109]. However, as with Ad11, Ad35 and other human subgroup B vectors, chimpanzee derived Ad vectors of subgroup B cannot be propagated on regular E1 producer cell lines. In this instance, rather than develop new producer cell lines, a chimeric vector approach is utilized. This strategy involves the central portion of the genome, which harbors several of the structural proteins to be derived from the subgroup B virus, but the flanking regions are from a non-subgroup B virus allowing these chimeric vectors to be propagated on HEK293 cells [110,117]. Furthermore, this strategy is similar to that of modifying the E4 region of human subgroup B viruses, to allow propagation in well characterized cell lines.

One of the interesting observations to arise from the use of producer cell lines for alternate Ad serotypes is that in many cases there was a shift from developing new producer lines towards further vector genome modifications. While it seemed necessary to generate specialized producer lines for the human subgroup B viruses, once their structure and biology was further delineated, it was realized that it is simpler to incorporate further vector modifications in the E4 region that allow for their propagation on a well-characterized E1 complementing cell line. This paradigm was also utilized with respect to simian based Ad vectors and is worth considering if there is rapid need to move a novel Ad vector from bench to bedside.

## 5. Conclusions

Throughout the last 3 decades a wide array of producer cell lines have been developed for the production of Ad vectors. There are still only a few cell lines approved for cGMP production of replication-defective Ad5 vectors, as the regulatory requirements include a very extensive process of cell banking and testing. As far as we know at the present time only HEK293, PER.C6, N52.E6, 293-ORF6 and 293FLP cell lines have approval for cGMP production of non-replicating Ad vectors, although unmodified A549 cells have been approved for cGMP production of replicating Ad vectors. While other laboratories develop additional cell lines, either as research tools to generate novel Ad vectors, to overcome RCA or licensing issues, regulatory considerations for cell lines should always factor into the terms of moving Ad vectors from bench to bedside in a time appropriate manner.

## Figures and Tables

**Table 1.** E1 complementary producer cell lines^a^.

**Cell Line**	**Parental Cells**	**Ad Sequence**	**Promoter Requirement**	**3′ End Requirement**	**Ref**
**HEK293**	Human embryonic kidney (HEK)	1 to 4344	Not applicable (N/A)	N/A	[2]

**911**	Human embryonic retinoblasts (HER)	79 – 5789	N/A	N/A	[5]

**pTG6559**	A549	505–4034	muPGK promoter	Rabbit β-globin gene polyA	[6]

**PER.C6**	HER	459–3510	huPGK promoter	Hepatitis B virus polyA	[7]

**GH329**	HeLa	511–3924	huPGK promoter	Yes	[8]

**N52.E6**	Primary human amniocytes	505–3522	muPGK promoter	SV40 splice acceptor and polyA	[9]

**HeLa-E1**	HeLa	542–3526	CMV	bGH polyA	[10]

**UR**	HEL 299	459–3510	RSV-LTR	TK polyA	[11]

**VLI-293^b^**	HEK293	1 to 4344, insertion of spacer at 3510	N/A	Hepatitis B virus polyA in insert	[12]

a These E1 producer cell lines contain contiguous E1A and E1B sequences. Two additional E1 producer cell lines, which have E1A and E1B inserted separately [13,14], are discussed in the body of text as an alternate method to this historical method.

b Although this cell line is novel, it still contains the contiguous E1A and E1B sequences with a Hepatitis B virus polyA at the 3′ end terminating E1B transcription.

**Table 2.** E1/E2 and E1/E4 complementary producer cell lines.

**Cell Line**	**Parental Cells**	**E2 or E4 Complementation**	**Viral Products**	**Inducible System**	**Refs**
**VL2-20 and VK10-9^a^**	HEK293	E4	All ORFs	Glucocorticoid	[31]
**293-E4**	HEK293	E4	All ORFs	cAMP	[49]
**C7**	LP-293	E2B	Pol and pTP^b^	No	[50,57]
**293-ORF6**	HEK293	E4	ORF6	Metal^c^	[51]
**MT-ORF6**	HEK293	E4	ORF6	Metal	[52]
**MMTV-ORF6**	HEK293	E4	ORF6	Glucocorticoid	[52]
**AE1-2a**	A549^d^	E2A	DBP	Glucocorticoid	[53]
**293-pTP**	HEK293	E2B	pTP	Tetracycline	[54]
**IGRP2**	HEK293	E4	ORF6 and 7	Glucocorticoid	[55]
**293-C2**	HEK293	E2A	DBP	No	[56]
**911E4**	911	E4	All ORFs	Tetracycline	[58]
**293-E2A**	HEK293	E2A	DBP	Glucocorticoid	[59]
**293-E4**	HEK293	E4	All ORFs	Yes^e^	[59]
**293-E4ORF6+7**	HEK293	E4	ORF6 and 7	Tetracycline	[59]
**A70.S54**	AE1-2a	E2A/E4	DBP/All ORF	Glucocorticoid	[60]
**E2T**	HEK293	E2A	DBP	Tetracycline	[61]

a These lines also express pIX under the control of metal inducible metallotheinen promoter.

b C7 cell line was first generated to express only Ad DNA polymerase [50] and then modified to express precursor terminal protein [57].

c Zinc-inducible.

d The AE1-2A cell line is based on A549 that also encodes E1 region (552 to 4090) under control of a minimal glucocorticoid-response element (GRE5) promoter.

e The E4 cassette was under control of the E1A inducible homologous E4 promoter.

**Table 3.** High capacity Ad vector producer cell lines using recombinase system.

**Cell Line**	**Parental Cells**	**Ad vector complementation**	**Recombinase System**	**Refs**
**293Cre4**	HEK293	E1	Cre recombinase	[69]
**293cre415^a^**	293Cre4	E1	Cre recombinase	[79]
**CRE8**	HEK293	E1	Cre recombinase	[70]
**CreE**	E2T	E1/E2A	Cre recombinase	[72]
**293FLP**	HEK293	E1	FLPe recombinase	[76]
**293CreFLP**	293Cre4	E1	either^b^	[76]
**293-FLPe6**	HEK293	E1	FLPe recombinase	[77]
**C7-Cre**	C7	E1/E2B	Cre recombinase	[73]
**116**	293N3S^c^	E1	Cre recombinase	[78]
**PER.C6-Cre**	PER.C6	E1	Cre recombinase	[75]

a Chen *et al*., 1996 [79] developed the cell line as described in the table, but Sandig *et al*., 2000 [71] utilized the cell line in a Cre/lox helper dependent Ad system. All other cell lines documented in the table have been developed by the groups referenced in the table.

b This cell line can be used with Cre or FLPe recombinase.

c This is a subclone of HEK293 that can be cultured as an adherent cell line or in suspension [81].
